# Predicting beta-turns in proteins using support vector machines with fractional polynomials

**DOI:** 10.1186/1477-5956-11-S1-S5

**Published:** 2013-11-07

**Authors:** Murtada Khalafallah Elbashir, Jianxin Wang, Fang-Xiang Wu, Lusheng Wang

**Affiliations:** 1 School of Information Science and Engineering, Central South University, Changsha, 410083, P.R. China; 2 Department of Mechanical Engineering, University of Saskatchewan, Saskatoon, Saskatchewan, S7N 5A9, Canada; 3 Department of Computer Science, City University of Hong Kong, 83 Tat Chee Avenue, Kowloon, Hong Kong; 4 Faculty of Mathematical and Computer Sciences, University of Gezira, Wad Madani, 20, Sudan

## Abstract

**Background:**

*β*-turns are secondary structure type that have essential role in molecular recognition, protein folding, and stability. They are found to be the most common type of non-repetitive structures since 25% of amino acids in protein structures are situated on them. Their prediction is considered to be one of the crucial problems in bioinformatics and molecular biology, which can provide valuable insights and inputs for the fold recognition and drug design.

**Results:**

We propose an approach that combines support vector machines (SVMs) and logistic regression (LR) in a hybrid prediction method, which we call (H-SVM-LR) to predict *β*-turns in proteins. Fractional polynomials are used for LR modeling. We utilize position specific scoring matrices (PSSMs) and predicted secondary structure (PSS) as features. Our simulation studies show that H-SVM-LR achieves Qtotal of 82.87%, 82.84%, and 82.32% on the BT426, BT547, and BT823 datasets respectively. These values are the highest among other *β*-turns prediction methods that are based on PSSMs and secondary structure information. H-SVM-LR also achieves favorable performance in predicting *β*-turns as measured by the Matthew's correlation coefficient (MCC) on these datasets. Furthermore, H-SVM-LR shows good performance when considering shape strings as additional features.

**Conclusions:**

In this paper, we present a comprehensive approach for *β*-turns prediction. Experiments show that our proposed approach achieves better performance compared to other competing prediction methods.

## Background

Secondary structure of proteins consists of basic elements; these elements are *α*-helices, *β*-sheets, random coils, and turns. *α*-helices and *β*-sheets are considered as regular secondary structure elements while the residues that correspond to turns structures do not form regular secondary structure elements. In turns structures the C*α*-atoms of two residues are separated by one to five peptide bonds and the distance between these C*α*-atoms is less than 7A°. The number of peptide bonds that separate the two end residues determines the specific turn type. In *α*-turns and *β*-turns, the two end residues are separated by four and three peptide bonds respectively. In *γ*-turns, *δ*-turns, and *π*-turns, the two end residues are separated by two, one, and five peptide bonds respectively. The most common types of turns structure that exist in protein are *β*-turns structure. They represent approximately 25% of the secondary structure of the proteins sequences. *β*-turns can reverse the direction of a protein chain therefore they are considered as orienting structure [1]. They also have significant effects in protein folding, because they have the ability to bring together and allow the interactions between the regular secondary structure elements. *β*-turns are not only important in protein folding but are also implicated in the biological activities of peptides as the bioactive structures that interact with other molecules such as receptors, enzymes and antibodies [2]. They are also important in the design of various peptidomimetics for many diseases [3]. Therefore, the prediction of *β*-turns is one of the important problems in molecular biology, which can provide valuable insights and inputs for the fold recognition and drug design.

There are different methods designed for *β*-turns prediction. These methods can be divided into statistical methods and machine learning methods. The statistical methods that are used in *β*-turns prediction include Chou-Fasman method [4], Thornton's algorithm [5], GORBTURN [6], 1-4 & 2-3 correlation model [7], sequence couple model [8], and COUDES method [9]. All of these statistical methods use the sequence as input except for COUDES, which is based on propensities and multiple alignments. COUDES also utilizes secondary structure predicted by PSIPRED [10], SSPRO2 [11], and PROF [12]. The machine learning methods include BTPRED [13], BetaTpred2 [14], MOLEBRNN [15] and NetTurnP [1], which are based on artificial neural networks (ANNs), Kim's method based on k-nearest neighbor (KNN) [16], as well as support vector machines (SVMs) based methods, which recently have become popular in the field of *β*-turns prediction. These SVMs based methods include BTSVM [17], Zhang and colleagues' method [18], Zheng and Kurgan's method [2], Hu and Li's method [19], the method of Liu et al. [20], DEBT [21], and the method of Tang et al. [22]. In BTBRED, secondary structure predictions are utilized with two layered network architecture. BetaTpred2 enhances the performance of *β*-turns prediction by using secondary structure prediction and evolutionary information in form of position specific scoring matrices (PSSMs) as input to the neural networks. MOLEBRNN uses PSSMs as input to a bidirectional Elman-type recurrent neural network. NetTurnP uses evolutionary information and predicted protein sequence features as input to two ANN layers whereas the first layer is trained to predict whether or not an amino acid is located in a *β*-turn. Kim's method encodes protein sequence using a window of up to 9 residues to be used as input to a KNN based method, which is combined with a filter that uses secondary structure predicted with PSIPRED for the central residue. In BTSVM, position specific frequent matrices (PSFMs) and PSSMs, both calculated with PSI-BLAST [23], are applied to encode input for SVM classifier. Zhang and colleagues' method is another SVM method that uses PSSMs over a 7-residue window and the secondary structure of the central residue predicted by PSIPRED as an input. In Zheng and Kurgan's method a SVM is utilized to predict *β*-turns using window based information extracted from four predicted secondary structures (PSSs) with a selected set of PSSMs as input to the SVM. The SVM based method developed by Hu and Li combines the increment of diversity, position conservation scoring function, and secondary structure predicted with PSIPRED to compute the inputs for prediction of *β*-turns and *γ*-turns. Liu et al. combine SVM with PSS information obtained by using E-SSpred, a secondary protein structure prediction method. DEBT predicts *β*-turns and their types using information from multiple sequence alignments, PSSs, and predicted dihedral angles. Tang et al. considered another type of one-dimensional string of symbols representing the clustered region of *ϕ*, *ψ *torsion pairs called shape strings as new features. In [24] we utilized the idea of under-sampling to create several balanced datasets. These balanced sets were used to train several SVMs classifiers independently. The SVMs were aggregated using a linear logistic regression model.

In this paper, we propose a new approach called H-SVM-LR (Hybrid approach of SVMs and Logistic Regression (LR)) for predicting *β*-turns. Our proposed approach incorporates the idea of clustering by partitioning the non-*β*-turn class into three subsets using k-means clustering algorithm. Each subset is merged with the positive class (*β*-turn) to form a sub training set. These sub training sets are used to train localized SVMs classifiers independently. LR model modeled using fractional polynomials, is used to aggregate the localized SVMs to make a collective decision. The merit of using LR to aggregate the localized SVMs is that it will enable us to take advantages of the statistical modeling theory to find the optimal weights for each local SVM [24]. Also LR has the advantages of being widely studied [25], and in the recent years there are many algorithms have been designed to improve its performance. These algorithms include iteratively re-weighted least squares (IRLS) algorithm, which is a special case of fisher's scoring method [26,27].

## Methods

### Support vector machine (SVM)

The SVM is a state-of-the-art supervised learning model with associated learning algorithm for analyzing and classifying data. It transfers the data from low dimensional space to high or infinite dimensional space and then construct a hyper-plane or hyper-planes in this higher dimensional space to classify the transformed data. Normally the training data are represented as points in a vector space. The hyper-plane with the largest distance to the nearest training data point is considered to be the good separator. Given a training set {*x_i_*, *y_i_*}_*i *= 1, ..., *l*_, where *x_i _*is a vector of features, and *y_i _*∈ {-1, 1}. SVM solves the following primal problem.

(1) $$min\frac{1}{2}||w|{|}^{2}+C\overset{l}{\underset{i=1}{\sum }}\xi_{i},$$

subject to

$$y_{i}\left(w.x_{i}+b\right)\geq 1-\xi_{i},\xi_{i}\geq 0,i=1,....,l,$$

where *w *is the normal vector to the hyper-plane, *b *is the offset from the origin, and *C *is the error penalty parameter. The kernel function, which maps the input space into a higher-dimensional space, can be applied to create SVM classifier for non-linear problem. The kernel functions that can be used for SVM include polynomial kernel function, radial basis (also known as Gaussian kernel function), and sigmoid kernel function.

### Logistic regression (LR)

LR is a type of regression analysis used for predicting the outcome of a variable that can take on a limited number of classes. A detailed description of logistic regression can be found in [25]. In brief, given input vectors *x_i _*∈ *R^n ^*and output values *y_i _*∈ {0, 1}, logistic regression can be fitted using the following likelihood to predict the probability of the output. This probability will be *p *if *y_i _*= 1, or 1 - *p *if *y_i _*= 0.

(2) $$L\left(\theta \right)=\overset{n}{\underset{i=1}{\prod }}{\left(p_{i}\right)}^{\left(y_{i}\right)}{\left(1-p_{i}\right)}^{\left(1-y_{i}\right)}$$

However, it is easier mathematically to work with log of equation. The log-likelihood, where the log will turn products into sums, can be defined as follows:

(3) $$lnL\left(\theta \right)=\overset{n}{\underset{i=1}{\sum }}\left(y_{i}lnp_{i}+\left(1-y_{i}\right)ln\left(1-p_{i}\right)\right)$$

The value of *θ *that maximizes *L*(*θ*) is called the maximum likelihood estimate and it is denoted as $\hat{\theta }$. For binary outputs, the loss function or the deviance (DEV) is the negative log-likelihood and is given by the following formula.

(4) DEV=-2lnL(θ)

Minimizing the deviance given in the above equation is equivalent to maximizing the log-likelihood.

### Datasets

The dataset BT426, which contains 426 non-homologous protein chains, is used to evaluate our H-SVM-LR prediction method. This dataset was developed by Guruprasad and Rajkumar [28]. We obtained it from Raghava Group's website http://www.imtech.res.in/raghava/bteval/dataset.html. The structure of protein chains in BT426 dataset is determined by X-ray crystallography at two resolution or better. In each chain there is at least one beta-turns structure. 24.9% of all amino acids in BT426 have been assigned to be having *β*-turns structure. Several recent beta-turns prediction methods use it as a golden set of amino acid sequences to evaluate their performances. We therefore used it to evaluate our methods and to make direct comparisons with the other prediction methods. Besides BT426, we used the dataset of 547 protein sequence (BT547), and the dataset of 823 protein sequence (BT823) to evaluate our approach. These datasets were constructed for training and testing COUDES [9].

### Features

#### PSSMs

It has been shown that PSSMs contributed significantly to the accuracy of *β*-turns prediction [1,2]. They are in the form of M*20, where M represents the sequence length. The PSSMs are generated using three rounds of the iterative PSI-BLAST program [23] against National Center for Biotechnology Information (NCBI) non-redundant (nr) sequence database with the default parameters. The PSSMs values are scaled to values between 0 and 1 using the following function.

(5) $$f\left(x\right)=\frac{1}{1+e^{-x}}$$

where *x *is the PSSM's element that stands for the likelihood of the particular residue substitution at that position.

#### Predicted secondary structure (PSS)

PROTEUS [29] is used to predict the secondary structure features. The motivation to use PROTEUS comes from the work of Tang et al. [22], which concludes that the predictions when using PROTEUS and PSSMs were better than when using PHD [30], JPRED [31], PROTEUS, and PSSMs together. The secondary structure features are predicted as three structure states: helix (H), strand (E) and coil (C). These three structure states are encoded as 1 0 0 for helix, 0 1 0 for strand, and 0 0 1 for coil.

#### Predicted shape strings

Tang et al. [22] predicted shape strings from a predictor constructed based on structural alignment approach. Shape strings were represented by eight states, i.e. S, R, U, V, K, A, T and G. They used a sliding window of 8 amino acids on PSSMs, PSS and shape strings features. We also added shape strings to our PSSMs and PSS features. The shape strings were predicted using the protein shape string and its profile prediction server (DSP) [32]. Besides the eight states DSP defines shape N where the *ϕ *and *ψ *angles are undefined, or no structure determination for parts of the sequence. The shape strings features are encoded as (1 0 0 0 0 0 0 0 0) for S, (0 1 0 0 0 0 0 0 0) for R, ..., and (0 0 0 0 0 0 0 0 1) for N.

### The proposed approach

**Figure 1:**
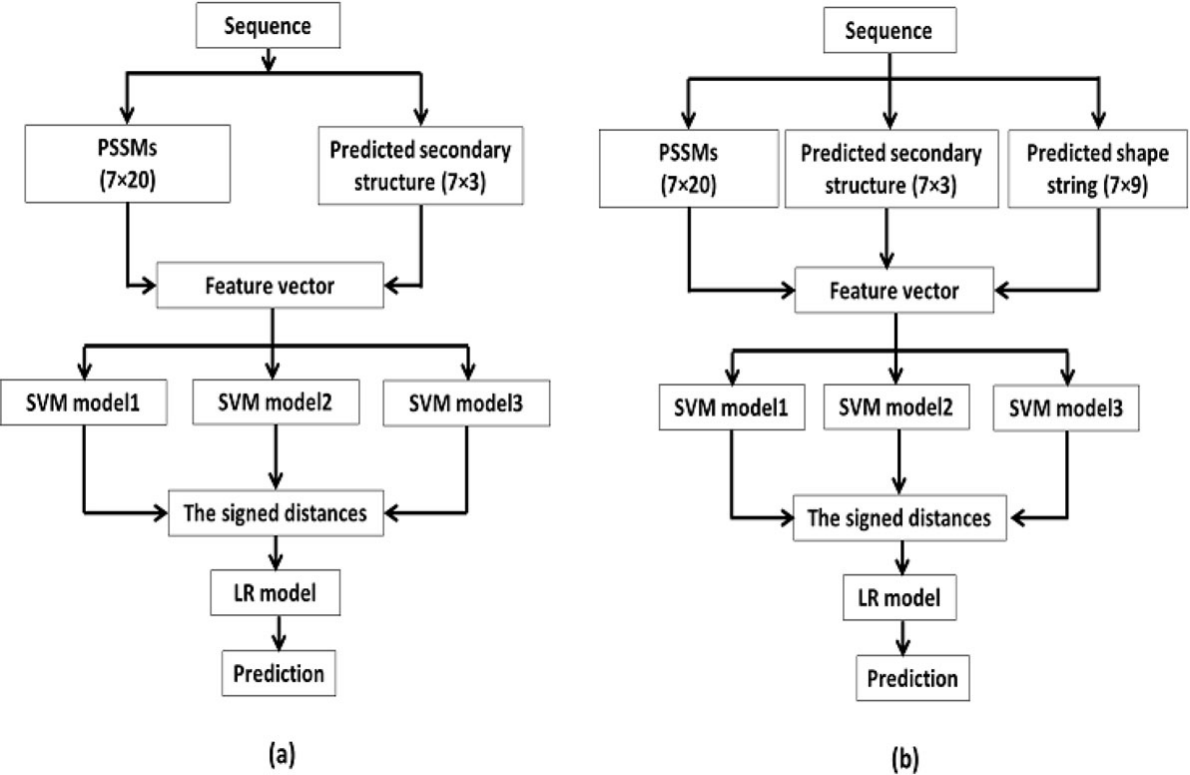
**The architecture of the proposed prediction method**. Figure 1(a) represents the prediction using PSSMs, and PSS, while Figure 1(b) represents the prediction using PSSMs, PSS, and shape strings. 7 denotes the window size, the PSSMs have 20 columns and there are 3 secondary structure states and 9 shape string states.

The entire framework of our proposed approach is shown in Figure 1. Three SVM classifiers are constructed using inputs from three clustered model. Then these three SVMs classifiers are integrated with logistic regression model. Statistical model selection based on fractional polynomials is used to take advantage of each classifier such that the final global classifier could have a better performance.

A sliding window of size seven residues is used over the matrix that consists of the features. The prediction is made for the central residue. This window size is selected in accordance with Shepherd et al. [13] who found that the optimal prediction for *β*-turns is achieved using window size of seven or nine.

### Clustered model

Since *β*-turns account for approximately 25% of the globular protein residues, the ratio of *β*-turns to non-*β*-turns is 1:3. Thus, the training sets used for *β*-turns prediction are imbalanced sets. In our trail experiments, we found that if the non-*β*-turns set is divided into a three subsets by a suitable clustering algorithm, each non-*β*-turns subset with the whole *β*-turns set will form approximately balanced training set. This balanced training set is more likely to be separable in the feature space. That is because the distribution of the non-*β*-turns samples in a subset is centralized and compacted. In other words, the *β*-turns set can be easily separated from each non-*β*-turns cluster by a different hyper-plane. That means good performance would be expected when constructing localized SVMs using each non-*β*-turns cluster against the *β*-turns. But, each of these SVMs alone is certainly not a good global classifier. It proposes that it is possible to construct a better classifier than the SVM trained with the whole data by combining these SVMs effectively. Particularly, a localized SVM classifier can be constructed for each sub training set, this way the localized SVMs will not be affected by the heterogeneity of the whole training set. To outperform the SVM that is trained with the whole data, we need to combine these localized SVMs effectively into global one without neglecting their local advantages. Majority voting is one of the methods that are used to combine several classifiers, but its main problem is that it will not give weight to each classifier. LR model can integrate the localized SVMs classifiers, and it allows us to take advantages of the statistical modeling theory to find the optimal weights for each local classifier. The motivation to use this clustered model comes from the work of Yi Chang [33]. In his work, Yi Chang used localized linear SVMs classifier for a data in the feature space defined by a chosen kernel.

**Figure 2:**
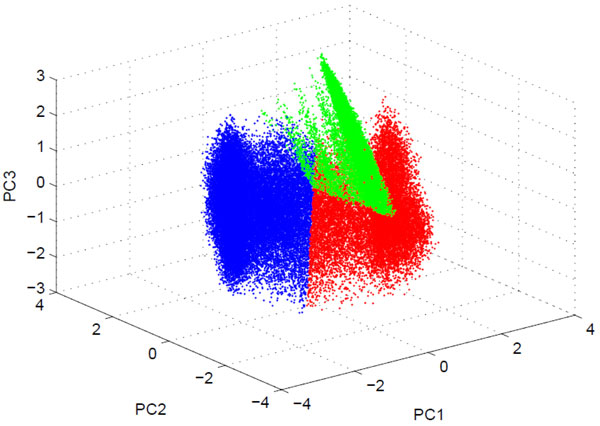
**The distribution of the three clusters**. The axes represents the top 3 PCs of principal component analysis (PCA) of negative samples (non-*β*-turns). Red dots denote samples in cluster 1, blue denotes samples in cluster 2, and green denotes samples in cluster 3.

At the very beginning, the whole negative examples are divided into three clusters by a *k*-means clustering algorithm using original variables. The distribution of those three clusters is shown in Figure 2. We merged the whole positive examples with each cluster to form three sub-training sets. These sub-training sets are used to build three SVMs models. The three SVMs will not be used directly in the prediction, but they will be used as variable generators. During training and prediction stages, these models are unchanged and all the samples enter all of the three models. The signed distance for each example to the separating hyper-planes of the three models is computed and stored in a vector *d *of dimension (N * 3), where *N *is the number of the instances. The vector *d *will be used as a new feature vector for a LR model, which will weigh the response of the three models and then calculates the prediction probability.

#### LR model selection

The components of the LR predictive model are obviously variables, which should be selected carefully so that the model makes accurate prediction, but without over-fitting the data. There are two competing goals in model selection. (1) It should be complex to fit the data well. (2) It should be simple to interpret. To select our LR model, we first looked at the correlation in the estimated coefficient. If two variables are highly correlated, we do not need both of them in the model. The uni-variate analysis was used to identify the important variables, in which the LR models with one variable at a time were fitted, and then the fits were analyzed. In particular, we looked at the estimated coefficients, their standard errors and the likelihood ratio test for the significance of the coefficients. Then we fitted our LR using the variables selected in the uni-variate analysis according to the following procedure:

- We verified the importance of each variable in the LR model using Wald statistics.

- We compared the coefficients of the each variable with the coefficient from the model containing only that variable.

- Any variable that did not appear to be important was eliminated, and a new model was fitted. The new model was checked whether it is significantly different from the old model. If it is, then the deleted variable is important.

- The process of deleting, refitting and verifying was repeated until it appears that all the important variables were included in the model.

- We tried to fit a linear LR model to the data but the prediction error is found to be very large, so we considered power transformation using fractional polynomials.

- A list of possible interactions between each pairs of variable was created, these interactions terms were added one at a time, in the model containing all the main effects and assess its significance using the likelihood ratio test. The significant interactions were added to the main effect model and its fit was evaluated using Wald tests and LR test for the interaction terms, and any non-significant interaction was dropped.

#### Fractional polynomials

The final outcome variable is the *β*-turn/non-turn response. In our hybrid model, this variable depends on the outcome of the three SVMs classifiers in a logistic regression model. The outcome of the three SVMs classifiers is represented by the vector *d *= (*d*_1_, *d*_2_, *d*_3_). The natural starting point, the straight line model *b*_0 _+ *b*_1_*d*_1 _+ *b*_2_*d*_2 _+ *b*_3_*d*_3 _or *b*_0 _+ *dB *in matrix form, where *B *is the vector of parameters, is first tested whether it is adequate. To improve the fit, we investigated other models. We looked for non-linearity by fitting a first order fractional polynomial to the data. The best power transformation $d_{i}^{p}$ was found, with the power *p *chosen from candidates -2, -1, -0.5, 0, 0.5, 1, 2, 3, where $d_{i}^{0}$ denotes *log*(*d_i_*). The set includes the straight line (i.e. no transformation) *p *= 1. The variables *d_i _*contain non-positive values, thus we transformed its values to values > 0, which will enable the use of logarithms and negative powers transformation. Including more powers usually offers only slight improvement in the model fit. In particular, there is a problem with including large negative powers, such as -3, that individual extreme observations will influence the fit too much [34]. The first-degree fractional polynomial provides unsatisfactory fit to our data, so we considered second-degree fractional polynomial. We used the closed test procedure, which first determine the best-fitting second degree polynomial by choosing the powers transformation *p *and *q *from the aforementioned set. For mathematical limit, when *p *= *q *for the variable *d_i _*in the model then the terms of the variable will be written in the form $b_{j}d_{i}^{p}+b_{k}d_{i}^{p}log\left(d_{i}\right)$. The best fit among the combinations of such powers is defined as that which maximizes the likelihood or equivalently that which minimizes the deviance [35]. The MFP package, which is a collection of R [36] functions targeted at the use of fractional polynomials for modeling the influence of continuous variables on the outcome in regression models is used in this research to find the best fit among the combinations of the powers *p *and *q*. MFP uses a sequential and a closed testing selection procedures for a single continuous variable. Using the BT426 dataset, our final model is selected after two cycles. The results of the model selection are shown in Table 1. The best-fit fractional polynomials (fractional polynomials with the lowest deviance) for SVM model1, SVM model2, and SVM model3 are underlined.

**Table 1:** Fractional polynomials for the SVMs models using the BT426 dataset.

	Cycle 1			Cycle 2		
		
Variable		Powers			Powers	
	Deviance			Deviance		
		P	q		P	q
SVM model1	256272.1			256255.1		
	256235.6	1		256209.8	1	
	256180.1	-0.5		256146.1	-0.5	
	256080.4	1	2	256035.3	1	2
SVM model2	257266.9			257050.1		
	256512.8	1		256314.3	1	
	256284.1	0		256086.0	0	
	256235.6	0.5	1	256035.3	0.5	1
SVM model3	258586.7			258511.7		
	256669.1	1		256247.5	1	
	256626.6	0.5		256148.6	0.5	
	256512.8	2	3	256035.3	2	2

### Training and testing

We used LIBSVM package [37] to train and build the SVMs prediction models. The radial basis kernel function was used to transfer the data from a low dimension space to a higher-dimensional space nonlinearly for all the SVMs. The default grid search approach was used to find the optimal values for the LIBSVM's parameters C and gamma. The leave-one-out cross-validation test, in which different datasets for training and testing are used to evaluate a prediction method, is an accurate test method compared with independent dataset test and sub-dataset test [38]. When using this test, one protein out of N proteins is removed to represent the testing set and the remaining N-1 proteins are combined together to represent the training set that will be used for training the prediction method. This process is then repeated N times by removing one protein in each time. In *β*-turns prediction, applying this process exactly is time consuming. Thus, most of the state-of-the-art *β*-turns prediction methods use seven-fold cross validation to assess their prediction performances [39]. Therefore, we used seven-fold cross validation to assess the performance of our H-SVM-LR method. We first started by dividing the dataset into seven subsets that contain equal numbers of proteins. In each set the *β*-turns account for approximately 25% of the protein residues, in other words each set contains the naturally-accruing proportion of beta-turns. We removed one set to represent the testing set and the other sets were merged together in one training set, which is used to train H-SVM-LR. This process was repeated seven times in order to have a different set for testing each time. We take the average of the results from the seven testing sets to represent the final prediction result.

### Performance measures

The quality of prediction is evaluated using four measures, the prediction accuracy, Qpredicted, Qobserved, and MCC. These measures are the most frequently used measures to evaluate the *β*-turns prediction methods. They are calculated using the four values (i) true positive (TP), which is the number of the residues that are correctly classified as *β*-turns, (ii) true negative (TN), which is the number of the residues that are correctly classified as non-*β*-turns, (iii) false positive (FP), which is the number of residues that have non-*β*-turns structure and incorrectly classified as having *β*-turns structure, and (iv) false negative (FN), which is the number of residues that have *β*-turns structure and incorrectly classified as having non-*β*-turns structure.

The prediction accuracy (also known as Qtotal) refers to the percentage of correctly classified residues and is calculated as follows:

(6) $$Qtotal=\frac{TP+TN}{TP+TN+FP+FN}\times 100$$

Qpredicted (also known as the predicted positive value (PPV) or the probability of correct prediction) refers to the percentage of the residues that are correctly predicted as *β*-turns among the predicted ones and is calculated as follows:

(7) $$Qpredicted=\frac{TP}{TP+FP}\times 100$$

Qobserved (also known as sensitivity or coverage) refers to the percentage of the residues that are correctly predicted to have *β*-turns structure among those observed as having *β*-turns structure. In other words, it represents the fraction of the total positive samples that are correctly predicted and it is calculated as follows:

(8) $$Qobserved=\frac{TP}{TP+FN}\times 100$$

Because of the imbalanced dataset (25% *β*-turns), Qtotal by itself is a poor measure. In other words, one can achieve a Qtotal of 75% (baseline accuracy) by predicting all the residues to be non-*β*-turns. Therefore, Matthew's correlation coefficient (MCC) [40] is an important, robust and reliable performance measure. The MCC can be obtained using the following formula:

(9) $$MCC=\frac{TP*TN-FP*FN}{\sqrt{\left(TP+FP\right)*\left(TP+FN\right)*\left(TN+FP\right)*\left(TN+FN\right)}}$$

Normally, the value of MCC is greater than or equal to -1 and less than or equal to 1. If the value of MCC is close to 1 then there is a perfect positive correlation, if it is close to -1 then there is a perfect negative correlation, and a value close to 0 indicates no correlation.

The receiver operating characteristic (ROC) curve is adopted in this paper as a threshold independent measure. The ROC curve provides the effectiveness of *β*-turns prediction method. The area under the ROC curve (AUC) is an important index that reflects the prediction reliability. A good classifier has an area close to 1, while a random classifier has an area of 0.5.

## Results and discussion

The methods that are applied on *β*-turns prediction use different PSSMs and PSS organizations. Some researchers use a sliding window on the PSSMs and then add the PSS e.g. [18]. Other researchers use a sliding window on both PSSMs and PSS e.g. [20]. Both ways are tested in our proposed method and the results for the BT426 dataset are shown in Table 2.

**Table 2:** Performance comparison between different features organization on the BT426 dataset.

Features organization	Qtotal	Qpredicted	Qobserved	MCC
A sliding window on PSSMs only	81.03	63.98	57.40	0.48
A sliding window on both PSSMs and PSS	82.87	64.83	70.66	0.56

**Figure 3:**
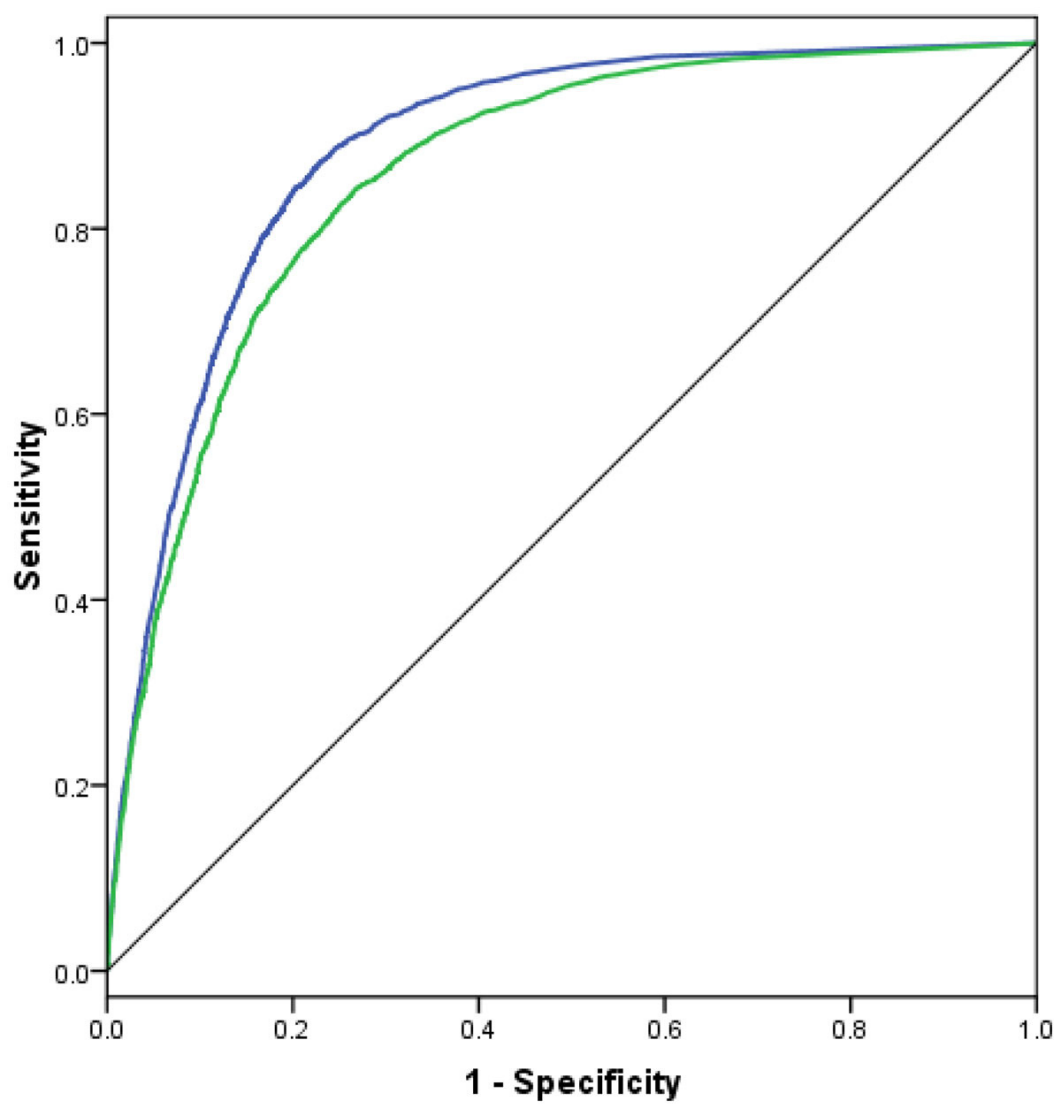
**ROC curves for the prediction using a sliding window on PSSMs only and sliding window on both PSSMs and PSS**. Blue curve corresponds to the prediction using sliding window on both PSSMs and PSS, while the green curve corresponds to the prediction using a sliding window on PSSMs only. The dataset used for drawing the curves is BT426.

From the results we found that the performance of H-SVM-LR using a sliding window on both PSSMs and PSS is by far better than using a sliding window on PSSMs only and then add the PSS for the central amino acid. Figure 3 shows the ROC curves for *β*-turns prediction using a sliding window on PSSMs only and a sliding window on both PSSMs and PSS. The AUC highlights the effect of using a sliding window on both PSSMs and PSS. The AUC value using a sliding window on both PSSMs and PSS is 0.89, 0.03 higher than using a sliding window on the PSSMs only.

Table 3 shows the comparison between H-SVM-LR and other existing *β*-turns prediction methods based on seven-fold cross validation on the BT426 dataset. H-SVM-LR achieves prediction accuracy or Qtotal = 82.87%, Qpredicted= 64.83%, Qobserved = 70.66%, and MCC = 0.56. The Qtotal of H-SVM-LR is the highest among the existing methods that use PSSMs and PSS as features; i.e. Zheng and Kurgan's method and the method of Liu et al. achieved Qtotal of 80.9. The difference in Qtotal between H-SVM-LR and these methods is 1.97%. We emphasize that this difference is relatively large when considering that the baseline accuracy equals to 75%, which could be obtained by merely regarding all residues as non-*β*-turns. i.e., H-SVM-LR provides 7.87/25 = 31.5% error rate reduction, while Zheng and Kurgan's method and the method of Liu et al. provide 5.9/25 = 24% error rate reduction, and Hu and Li's method provides 4.8/25 = 19% error rate reduction.

**Table 3:** Comparison of H-SVM-LR with other *β*-turns prediction methods on the BT426 dataset.

Prediction method	Qtotal	Qpredicted	Qobserved	MCC
H-SVM-LR	82.87	64.83	70.66	0.56
Zheng and Kurgan [2]	80.9	62.7	55.6	0.47
Liu et al. [20]	80.9	63.6	49.2	0.44
Hu and Li [19]	79.8	55.6	68.9	0.47
DEBT [21]	79.2	54.8	70.1	0.48
BTSVM [17]	78.7	56.0	62.0	0.45
NetTurnP [1]	78.2	54.4	75.6	0.50
MOLEBRNN [15]	77.9	53.9	66.0	0.45
Zhang et al.(multiple alignment) [18]	77.3	53.1	67.0	0.45
BetaTPred2 [14]	75.5	49.8	72.3	0.43
Kim [16]	75.0	46.5	66.7	0.40
COUDES [9]	74.8	48.8	69.9	0.42
BTPRED [13]	74.4	48.3	57.3	0.35

a

Note: The results of the method of Liu et al. and NetTurnP method are obtained from their corresponding papers. The results of other *β*-turns prediction methods are obtained from [22].

H-SVM-LR shows high MCC 0.56 compared to NetTurnP 0.50, Zheng and Kurgan's method 0.47, and the method of Liu et al. 0.44. Thus, H-SVM-LR has the highest MCC and Qtotal among the other *β*-turns prediction methods. The MCC value achieved is noteworthy since MCC accounts for both over predictions and under predictions. The Qobserved of H-SVM-LR is higher by 15.06% than the Qobserved of Zheng and Kurgan's method, by 1.76% than the Qobserved of Hu and Li's method, and by 21.46% than the Qobserved of the method of Liu et al. Higher Qobserved values mean that a large percentage of the observed *β*-urns is correctly predicted. At the same time, the Qpredicted of our method shows that more than 64% of the actual *β*-turns are correctly predicted. We note that the Qpredicted of H-SVM-LR is 2.13% higher than the Qpredicted of Zheng and Kurgan's method, by 9.23% than the Qpredicted of Hu and Li's method, and by 1.23% higher than the Qpredicted of the method of Liu et al.

Besides BT426 dataset that is used for training and testing H-SVM-LR, we used two additional datasets, i.e. BT547 and BT823 datasets, to validate its performance. Results obtained based on seven-fold cross validation on these datasets are given in Table 4. The results show that for the BT547 dataset H-SVM-LR obtains Qtotal = 82.84%, Qpredicted = 63.60%, Qobserved = 68.50%, and MCC = 0.55. The MCC and Qtotal of H-SVM-LR are the best among the other competing methods that are evaluated on BT547 dataset. We note that the Qpredicted of H-SVM-LR is 0.7% lower than the Qpredicted of the method of Liu et al., while the Qobserved of H-SVM-LR is 24% higher than the Qobserved of the method of Liu et al. The increase in the Qobserved value is a trade-off for the decrease in the Qpredicted value. In spite of this trade off, H-SVM-LR shows high overall accuracy. For the BT823 dataset H-SVM-LR obtains Qtotal = 82.32%, Qpredicted = 64.48%, Qobserved = 72.72%, and MCC = 0.56. Also H-SVM-LR has the highest MCC, Qtotal, Qpredicted, and Qobserved on BT823 datasets. The results also show that H-SVM-LR shows stable performances on all the three datasets used. Note that we used the same LR model that is used for testing BT426. These results indicate that H-SVM-LR can better discriminate between *β*-turns and non-*β*-turns.

**Table 4:** Comparison of H-SVM-LR with other *β*-turns prediction methods on BT547 and BT823 datasets.

Prediction method	Dataset	Qtotal	Qpredicted	Qobserved	MCC
H-SVM-LR		82.84	63.60	68.5	0.55
Zheng and Kurgan [2]		80.5	61.6	54.2	0.45
Liu et al. [20]	BT547	80.6	64.3	44.5	0.44
Hu and Li [19]		76.6	47.6	70.2	0.43
DEBT [21]		80.0	55.9	68.7	0.49
COUDES [9]		74.6	48.7	70.4	0.42

H-SVM-LR		82.32	64.48	72.72	0.56
Zheng and Kurgan [2]		80.6	60.8	54.6	0.45
Liu et al. [20]	BT823	80.5	62.3	44.6	0.44
Hu and Li [19]		76.8	53.0	72.3	0.45
DEBT [21]		80.9	55.9	66.1	0.48
COUDES [9]		74.2	47.5	69.6	0.41

a

Note: The results of the method of Liu et al. are obtained from their corresponding paper. The results of other *β*-turns prediction methods are obtained from [22].

### Including shape strings features

**Figure 4:**
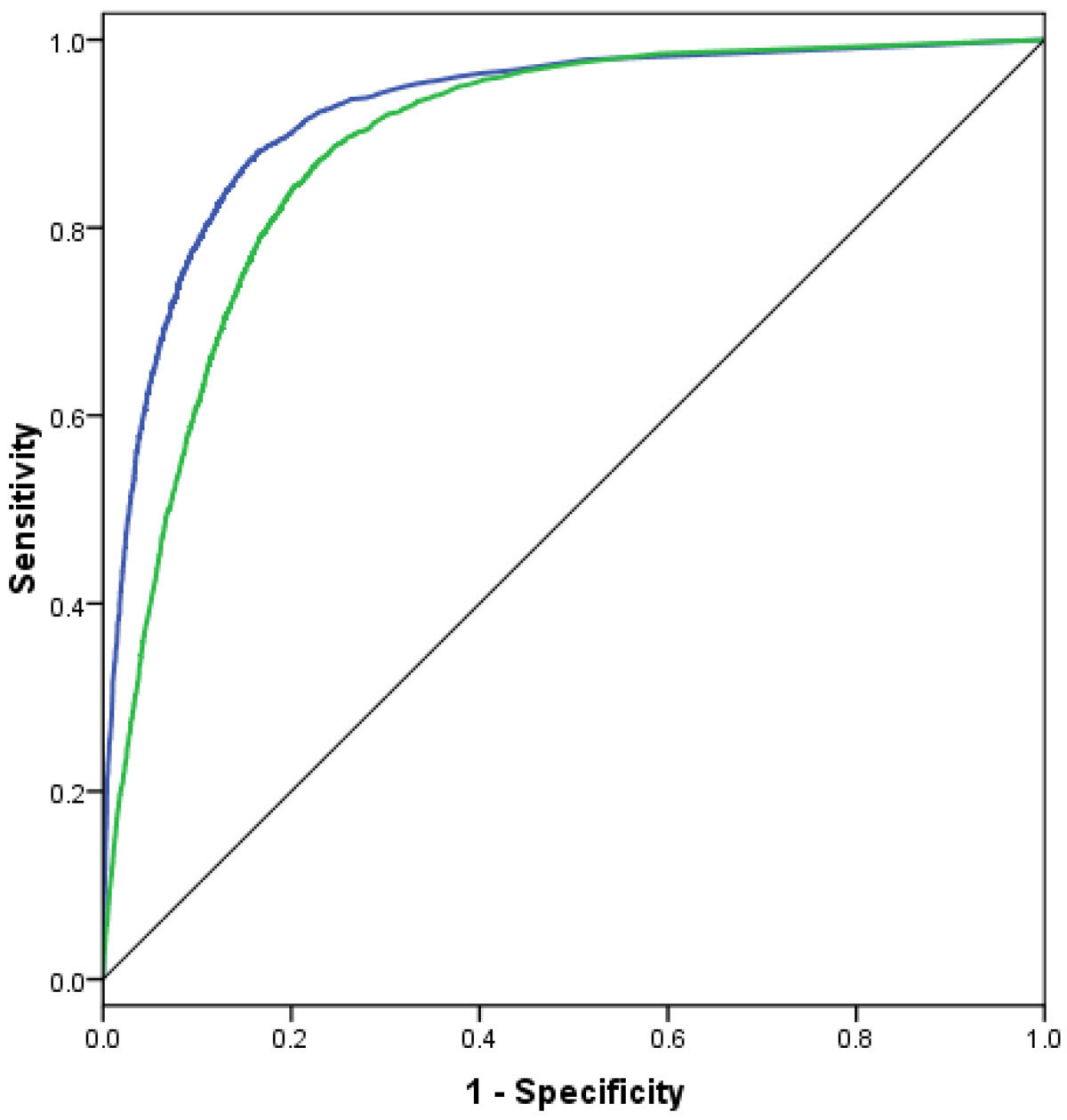
**ROC curves for the prediction before and after including the predicted shape strings**. Blue curve corresponds to the prediction after including the predicted shape strings, while the green curve corresponds to the prediction before including the predicted shape strings. The dataset used for drawing the curves is BT426.

The comparisons between H-SVM-LR after including the shape strings features and the method of Tang et al. on the BT426, BT547, and BT823 are shown in Table 5. Figure 4 depicts the ROC curves for *β*-turns prediction using H-SVM-LR before and after adding the shape strings for the BT426 dataset. The AUC value when including the shape strings is 0.923, while the AUC value when using PSSMs and PSS only is 0.886.

**Table 5:** Comparison of H-SVM-LR with the method of Tang et al. [22].

Prediction method	Dataset	Qtotal	Qpredicted	Qobserved	MCC
H-SVM-LR	BT426	87.37	74.99	75.20	0.67
Tang et al.		87.2	73.8	75.9	0.66

H-SVM-LR	BT547	88.64	77.79	76.31	0.70
Tang et al.		87.3	69.8	86.5	0.69

H-SVM-LR	BT823	89.55	79.53	77.73	0.72
Tang et al.		88.7	72.6	88.1	0.73

## Conclusions

In this paper, we proposed an approach that combines SVM and LR to create a hybrid method for *β*-turns prediction. We called this hybrid method H-SVM-LR. In H-SVM-LR, we utilized protein profile in the form of PSSMs, and PSS as features. We also considered shape strings as additional features. We divided the non-*β*-turn class into three partitions using *k*-means clustering algorithm and then each partition is combined with the *β*-turn class to form approximately balanced sub-training sets. SVM classifier is used for each sub-training set. Using this procedure, the problem of imbalanced class can be overcome, and the SVM computational time can be reduced. LR model selected based on fractional polynomials is used to aggregate the decisions of the SVMs to come up with final *β*-turn or non-*β*-turn decision. Using LR to aggregate the decisions of the SVMs enables us to take advantages of the statistical modeling theory to find the optimal weights for each SVM. H-SVM-LR achieved MCC of 0.56, and Qtotal of 82.87% on the BT426 dataset when using PSSMs and PSS as features. The MCC and the Qtotal achieved are significantly higher than the best existing methods that predict beta-turns using PSSM and PSS. Also H-SVM-LR obtained the highest MCC and Qtotal on BT547 and BT823 datasets. Furthermore, H-SVM-LR shows good performance when including shape strings features.

## Competing interests

The authors declare that they have no competing interests.

## Authors' contributions

MKE computed the features, generated the prediction model, performed experimental comparison and drafted the manuscript. JW, FW, LW participated in the design of the study and helped to draft the manuscript. All authors have read and approved the final manuscript.

## Declarations

The publication costs for this article were funded by the corresponding author.

This article has been published as part of *Proteome Science *Volume 11 Supplement 1, 2013: Selected articles from the IEEE International Conference on Bioinformatics and Biomedicine 2012: Proteome Science. The full contents of the supplement are available online at http://www.proteomesci.com/supplements/11/S1.
